# Strong Conscious Cues Suppress Preferential Gaze Allocation to Unconscious Cues

**DOI:** 10.3389/fnhum.2018.00427

**Published:** 2018-10-16

**Authors:** Andrea Alamia, Oleg Solopchuk, Alexandre Zénon

**Affiliations:** ^1^Institute of Neuroscience, Université catholique de Louvain, Brussels, Belgium; ^2^UMR5287 Institut de Neurosciences Cognitives et Intégratives d’Aquitaine (INCIA), CNRS, Bordeaux, France

**Keywords:** unconscious learning, eye movements, visual attention, eye tracking, implicit learning

## Abstract

Visual attention allows relevant information to be selected for further processing. Both conscious and unconscious visual stimuli can bias attentional allocation, but how these two types of visual information interact to guide attention remains unclear. In this study, we explored attentional allocation during a motion discrimination task with varied motion strength and unconscious associations between stimuli and cues. Participants were instructed to report the motion direction of two colored patches of dots. Unbeknown to participants, dot colors were sometimes informative of the correct response. We found that subjects learnt the associations between colors and motion direction but failed to report this association using the questionnaire filled at the end of the experiment, confirming that learning remained unconscious. The eye movement analyses revealed that allocation of attention to unconscious sources of information occurred mostly when motion coherence was low, indicating that unconscious cues influence attentional allocation only in the absence of strong conscious cues. All in all, our results reveal that conscious and unconscious sources of information interact with each other to influence attentional allocation and suggest a selection process that weights cues in proportion to their reliability.

## Introduction

Attention is a mechanism for allocating cognitive resources to relevant stimuli (Desimone and Duncan, 1995). The highest priority for allocating attention is thought to be associated to the stimulus that maximizes expected information gain (i.e., provide the most information given the stimuli and its context; Summerfield and Egner, 2009; Manohar and Husain, 2013; Vossel et al., 2014). Attentional selection can be driven by stimulus- and context-specific features that make a given visual object stand out from its surrounding (Itti, 2005). This type of attention has been coined stimulus-driven, exogenous or bottom-up attention (Filali-Sadouk et al., 2010). On the other hand, selective attention can be driven to stimuli, features, or spatial location which are especially relevant with respect to the task that we are performing, an instance called goal-directed or top-down attention (Baluch and Itti, 2011; Henderson and Hayes, 2017).

Most studies of goal-directed attention have focused on attentional allocation to conscious cues (e.g., blue stimuli when looking for a blue flower). However, the influence of unconscious processing on visual attention remains debated. Despite persistent skepticism regarding the existence of unconscious processes (Shanks and St. John, 1994; Vadillo et al., 2016), many studies have reported evidence of unconscious processing in perceptual decision making (Greenwald et al., 1995; Peremen and Lamy, 2014; Alamia et al., 2016), motor learning (Cleeremans, 1993; Destrebecqz et al., 2005; Clerget et al., 2012) and dynamic system control (Berry and Broadbent, 1988). Regarding its interaction with attentional mechanisms, a recent study from Zhao et al. (2013) provided evidence in support of preferential attentional allocation to unconscious cues by showing that reaction times (RTs) were faster when task stimuli were presented within a structured stream of stimuli. In line with these results, in a previous study from our group, we also showed that temporal statistical regularities affect overt attention (i.e., eye movements): participants were attending more frequently the predictable onset of a novel target. Interestingly, we found this effect exclusively when the predictability was task-relevant (Alamia and Zénon, 2016 ). Another instance of unconscious influence on attentional allocation can be found in contextual cueing (Chun and Jiang, 1998; Chun, 2000), in which participants perform a visual search between two sets of stimuli having two different colors, only one of which includes the target. Unbeknown to participants, target location is paired with the spatial configurations of either one or the other set of stimuli. Even though the truly unconscious nature in this paradigm has been called into question (Smyth and Shanks, 2008), the results show that participants implicitly learn the rule, and bias their eye movements toward the portion of space where the target is more likely to appear (Jiang and Chun, 2001). In line with these results, studies based on subliminal spatial cues attained similar conclusions (Mulckhuyse et al., 2007; Mulckhuyse and Theeuwes, 2010). Thus, in the visual domain, evidence seems to suggest that attention is affected by predictability, even though previous studies have not tested subjects’ awareness thoroughly (Shanks and St. John, 1994; Newell and Shanks, 2014).

In ecological scenarios, conscious and unconscious sources of information are often mingled together, but how attentional allocation to unconscious cues interact with the amount of conscious information in the stimulus (i.e., signal strength) has not been investigated thoroughly so far. Here, we address this question aiming specifically at: (1) confirming that overt attentional exploration is influenced by unconscious sources of information, using a paradigm that addresses the main criticisms formulated against previous studies of unconscious processing; and (2) testing whether and how conscious and unconscious information interact when influencing attention. We explored these questions by exploiting an experimental design that leads to robust unconscious learning, in which color information biases decisions in a motion discrimination task and in which the awareness of the association has been thoroughly tested (Alamia et al., 2016). We manipulated the strength of the conscious signal (i.e., how easy it is to perceive the dots’ motion) to investigate how the reliability of conscious cues affects the weight of unconscious sources of information on attentional allocation. We hypothesized that: (1) eye movements are influenced by unconscious cues, in agreement with previous findings; and (2) reliable conscious information should decrease the influence of unconscious cues on task performance and attentional allocation.

## Materials and Methods

### Participants

Twenty-two healthy participants (15 females, mean age = 23.17, std = 1.68) took part in this experiment, receiving monetary compensation for their participation. All of them reported normal or corrected-to-normal vision. Two participants were discarded from the analyses because, during the debriefing at the end of the experiment, they could explicitly verbalize the association between color and motion. Therefore, all subsequent analyses were performed on 20 participants (the target sample size was estimated from previous experiments using similar task; Alamia et al., 2016). All the participants signed a written informed consent before the experiment. The experiment was approved by the local Ethics committee of the Université Catholique de Louvain and was carried out in accordance with the Declaration of Helsinki.

### Experimental Design

**Figure 1:**
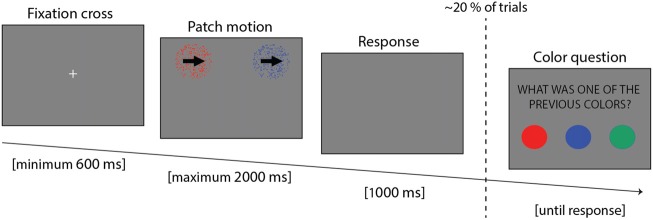
Trial example showing the three parts: fixation cross, stimulus presentation and response time.

Participants were comfortably made to sit, with the head placed on a chin rest, at a distance of 58 cm from the screen. Eye movements and blinks were recorded with an Eyelink^©^ 1,000 + eye tracker (SR Research Ltd., Kanata, ON, Canada; sample rate of 500 Hz). The experiment was implemented in Matlab 7.5 (The MathWorks, Natick, MA, USA), using the version 3.0.9 of the Psychotoolbox (Brainard, 1997). The experiment lasted around 45 min, and it was composed of 14 blocks, each lasting 56 trials. Each trial consisted of three parts (Figure 1): at first a fixation cross was displayed until the participants maintained fixation continuously for 600 ms; then two motion patches (see below) were presented until participants provided an answer, but no longer than 2,000 ms; finally, if the participant failed to provide an answer during the response period, an additional 1,000 ms blank display was presented to allow more time to respond.

An auditory feedback was provided to inform the participants of the accuracy of their response. The stimuli consisted of two patches of dots, each having a diameter of 6° and located at two of the four pseudo-randomly selected corners of the screen, 20° from the center (center to center distance). The motion of the two patches of dots was either rightward or leftward, and both patches always had the same motion direction. Participants were instructed to fixate the cross at the beginning, and then visually explore the two patches to report their motion direction. The patches could have two different coherence levels: 25% and 60%. The coherence level of a patch reflects the percentage of dots moving towards the main direction of motion (i.e., left/rightward). The motion direction of the remaining dots was selected at random. The lower the coherence the more difficult it is to discriminate the motion direction (Gold et al., 2008). Thus, there were three types of trials: easy (both patches at 60% coherence), difficult (both patches having 25% coherence) and mixed (one patch 60% and the other 25%). The rationale for having two levels of coherence was two-fold. On the one hand, it was used to investigate the interaction between conscious and unconscious sources of information on attentional allocation. On the other hand, it was also used as a way to favor visual exploration, since in pilot studies that did not include this manipulation, we found that subjects tended to fixate stimuli on the basis of their spatial location. In addition to coherence, the overall difficulty level of the task was tuned subject by subject in the first six blocks by changing the lifetime of the dots of both patches. Dot lifetime corresponds to the number of frames each dot is displayed before disappearing: the longer the lifetime, the easier it is to perceive the motion. The possible lifetime values were 15, 6, 4, 3 and 2, chosen on the basis of pilot studies performed on a different set of participants (given 100 Hz refresh rate, each frame lasts 10 ms and a lifetime of two corresponds to 20 ms). On the first training block, the lifetime of both patches was 15, and it was decreased by one level in subsequent blocks provided the participants’ average performance of the last block was above 70%. This approach allowed us to adjust motion discrimination difficulty to each participant, while concomitantly training them on the task. All except one participant reached the shortest lifetime (i.e., lifetime of 2) before the 7th block. Starting from the 7th block, the lifetime remained unchanged throughout the whole experiment. All the dots of each patch were of the same color, with three possible colors (i.e., red, blue and green). Unbeknownst to the participants, starting from the 7th block, one color was always associated to the rightward direction, another color to the leftward direction and the last color was uninformative of the motion direction of the dots. This association was pseudo randomized between participants. Briefly, two types of trials were possible: both patches shared the same color, or they had different colors. The color associated with leftward motion and the one associated with rightward motion were never presented together (the color-motion association was 100% valid). The frequency of occurrence of the colors was balanced during the whole experiment. Moreover, in 20% of the trials (11 out of 56 in a block chosen randomly and independently of the conditions) participants were asked to report one of the patch colors, forcing them to pay attention to the colors and providing us with an additional measure of the attended color. When the patches had different colors, both answers were considered as correct. All the possible types of trials are summarized in Table 1 (see in “Data Analysis” section). At the end of the experiment, participants responded to a de-briefing questionnaire composed of four questions: first, whether one motion direction was easier to discriminate than the other; second, whether one of the four positions was attended more than the others; third, whether the motion was easier to perceive with one of the three colors; and fourth, whether they had remarked an association between colors and motion. In case of positive answer to this last question, they were asked to report the nature of the association.

**Table 1:** All possible conditions (*n* represents the number of trials per condition per block).

	Patch 1		Patch 2	
	Coherence	Color	Coherence	Color
Condition 1 (*n* = 4)	60% (easy)	predictive	60% (easy)	non-predictive
Condition 2 (*n* = 4)	25% (difficult)	predictive	25% (difficult)	non-predictive
Condition 3 (*n* = 16)	60% (easy)	predictive	25% (difficult)	predictive
Condition 4 (*n* = 4)	60% (easy)	non-predictive	25% (difficult)	non-predictive
Condition 5 (*n* = 4)	60% (easy)	predictive	25% (difficult)	non-predictive
Condition 6 (*n* = 4)	25% (difficult)	predictive	60% (easy)	non-predictive
Condition 7 (*n* = 20)	Same as patch 2		Same as patch 1	

*Only the first four conditions have been considered for the eye-movement analyses, whereas condition 7—in which the two patches were identical—was used in the first behavioral analysis. Note that condition 7 is actually composed of four sub-conditions, i.e., when both patches are: easy-predictive, easy-unpredictive, difficult-predictive or difficult-unpredictive. Each sub-condition consists of five trials per block*.

### Data Analysis

During the whole experiment, we recorded eye position, participants’ responses and RTs. Two participants out of 22 reported the correct color-motion association in the final questionnaire, and thus were excluded from further analyses.

Statistical analyses consisted of Bayesian ANOVA, performed in JASP (Love et al., 2015): all the Bayes Factors, if not otherwise specified, refer to the alternative hypothesis, and are reported as BF_10_. Practically, a BF between 0.3 and 3 advocates for a lack of effect, whereas BF below 0.3 or above 3 suggests evidence in favor of the null or alternative hypothesis, respectively. The larger the BF, the stronger the evidence in favor of the alternative hypothesis (Bernardo and Smith, 2001; Masson, 2011). All other analyses, including eye movement preprocessing and feature extraction were performed in Matlab7.5 (The MathWorks, Natick, MA, USA).

#### Accuracy and RT Analysis

We performed behavioral and eye-movements analyses starting from the 7th block, i.e., the block in which the color-motion association was introduced in the experiment. Regarding the behavioral part, we tested two Bayesian ANOVAs considering either the accuracy (model I) or RT (model II) as dependent variables: the accuracy was modeled as a binary variable, whereas the RT was modeled as Gaussian. The independent variables considered were: PREDICTABILITY (a categorical variable modeling whether the color was informative or not), BLOCKS (categorical variable from 7 to 14), DIFFICULTY (categorical variable modeling whether the trial was difficult or easy) and all their interactions. Initially, we analyzed only trials in which the patches had the same color and the same coherence level, in order to remove all confounds induced by eye movements during patch selection (condition 7 in Table 1). In a second analysis, to study how selective attention affected accuracy and RT, we performed two additional Bayesian ANOVAs, one on accuracy (model III) and one on RT (model IV), considering the patch on which attention was allocated. We determined attention allocation as the distance between the eye position and the patches at the time of the participant’s response (see “Eye Movement Analysis” section below). Trials in which participants moved back and forth between the patches were excluded from this analysis (~15% of the trials). As in the previous analysis, we considered PREDICTABILITY and DIFFICULTY of the attended patch as factors. In this analysis and in the subsequent analyses regarding eye movements, we focused on two sub-categories of trials in order to simplify the interpretation of the findings (see Table 1): the first category of trials (conditions 1 and 2 in Table 1) included patches with the same coherence but different predictability levels (one predictive and one non-predictive). In the second category, patches had the same predictability in the color-motion association but different coherence levels (conditions 3 and 4 in Table 1). This approach allowed us to investigate specifically the effects of the color-motion association (condition 1 and 2), independently from the effect of difficulty (i.e., coherence levels, condition 3 and 4) on attentional allocation. We did not investigate further the remaining conditions 5 and 6 because it would have been challenging to properly disentangle the effects of predictability from the effect of patch coherence.

#### Eye Movement Analysis

We first removed the blinks (automatically detected by the Eyelink^©^ algorithm) by means of linear interpolation. Afterward, we determined attentional allocation trial by trial, on the basis of which patch was attended by the participants when they provided the answer: first we computed the distance between the eye position and each patch, then we normalized these distances by their sum, and finally we attributed a positive value in the attentional allocation binary variable to the patch with a normalized distance lower than 0.4. We then compared the percentage of trials in which the predictive or non-predictive patches were attended (model Va, conditions 1 and 2) and the easy or the difficult one was attended (model Vb, conditions 3 and 4). In this analysis, we included only trials in which participants attended a single patch (around 85% of all the trials). In a second analysis, we focused on the remaining portion of trials in which participants switched their attention from one patch to the other (around 15% of the trials): we compared the percentage of time in which they switched from predictive to non-predictive color and vice versa (model VIa, conditions 1 and 2) and from easy to difficult patches and vice versa (model VIb, conditions 3 and 4). Both models V and VI were implemented by means of Bayesian ANOVA. Finally, as an indirect measure of attention, we tested whether participants reported more often the predictive or the non-predictive color, when asked, at the end of each trial, which colors had been presented (Bayesian paired *t*-test).

## Results

### Behavioral Analysis

**Figure 2:**
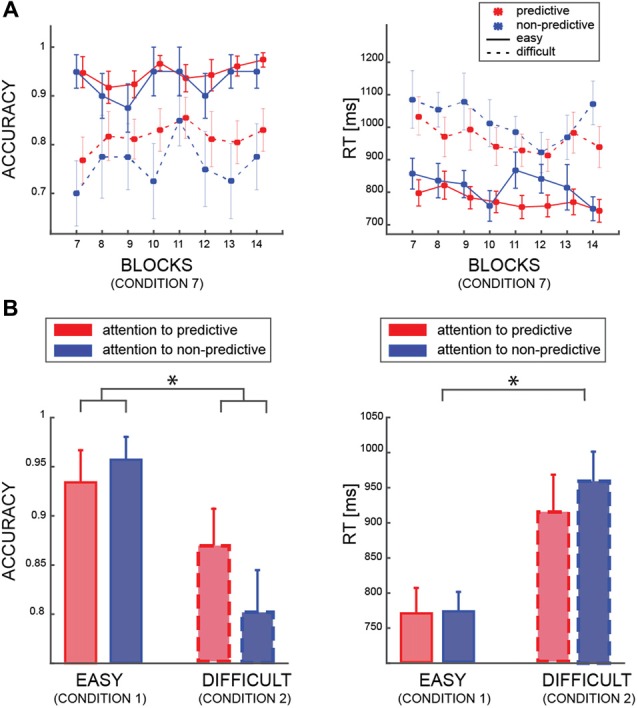
**(A)** Averaged accuracy and reaction time (RT) results for the easy (solid lines) and difficult (dashed lines) trials, and for predictive (red) and non-predictive (blue) trials. All data are from trials in which the patches are identical (condition 7—see Table 1). **(B)** Averaged accuracy and RT results according to which patch was attended by participants (same color code as in “a”) in trial from condition 1 and condition 2 (see Table 1). In all panels, error bars are standard errors, and asterisks indicate significant difference (*p* < 0.05).

In the first model (model I), we tested whether accuracy was affected by the PREDICTABILITY of the color, the BLOCK and the DIFFICULTY factors. This analysis was restricted to trials in which both patches had the same color and coherence level (i.e., condition 7, see Table 1). As expected, we found a very strong effect of the factor DIFFICULTY (BF_10_ ≫100 very strong evidence), indicating that participants were better at discriminating the motion direction of the patches when both had a coherence of 60%, than when both had 25% coherence (Figure 2A). Interestingly, we found also an effect of the factor PREDICTABILITY (BF_10_ = 17.86, strong evidence), with better performance for predictive than non-predictive colors, confirming that participants learned the implicit association between color and motion. No interaction between PREDICTABILITY and DIFFICULTY was found (BF_inclusion_ < 1). We found a strong negative results of the factor BLOCK (BF_01_ = 42.08) but all related interactions lacked sensitivity (all 0.3 < BF_10_ < 3).

Regarding RT (model II), we found a similar significant effect of DIFFICULTY (BF_10_ ≫ 100 very strong evidence), indicating faster responses for easier patches (i.e., patches with 60% coherence), and an effect of PREDICTABILITY (BF_10_ ≫ 10 strong evidence). All the other factors or interactions, were far from reaching significance (all 0.3 < BF_10_ < 3).

The second behavioral analysis aimed at investigating how accuracy and RT changed as a function of attentional allocation (Figure 2B). Here, we included only trials in which coherence was identical in both stimuli, such that the patches differed only in color (i.e., conditions 1 and 2, see Table 1). We found that the difficulty level of the attended patch affected performance (Model III; BF_10_ ≫ 100, very strong evidence), but we found no main effect of the ATTENDEND_PREDICTABILITY factor (BF_10_ = 1.2). Nevertheless, we found positive evidence of an interaction between DIFFICULTY and ATTENDEND_PREDICTABILITY (BF_10_ = 3.425), contrary to the results from condition 7 (i.e., both patches are identical—no interaction). This analysis reveals that participants were exploiting the predictability when one patch but not the other was predictive, and specifically when both patches were difficult (*post hoc* comparison between predictive and non-predictive patches in the difficult condition: BF_10_ = 3.824, in the easy condition BF_10_ = 0.410). Regarding the RT (model IV), we also found a significant impact of the factor DIFFICULTY (BF_10_ ≫ 100, very strong evidence) but no other effects (all 0.3 < BF_10_ < 3). All in all, these analyses show that participants’ accuracy was higher when they attended the patch whose color conveyed information about motion direction when the motion of both patches was harder to perceive.

### Questionnaire Analysis

We provided a questionnaire with four questions at the end of the experiment, with the purpose of assessing the awareness of the associations. The last question asked directly whether participants had remarked a color-motion association, and in case of a positive answer they were invited to specify which one. Only 2 out of 22 participants were able to provide the correct association (the remaining 20 participants responded negatively and did not provide any associations). The precedent three questions about general biases (see “Materials and Methods” section for details) were meant to keep the participants unaware of the true purpose of the questionnaire, thus not implying the existence of the association. The responses to these previous questions did not reveal any specific bias in the participants (specifically, five and three participants reported respectively rightward and leftward motion direction as easier to perceive; and three and two participants reported respectively upper and lower positions as easier to discriminate motion directions).

### Eye Movement Analysis

**Figure 3:**
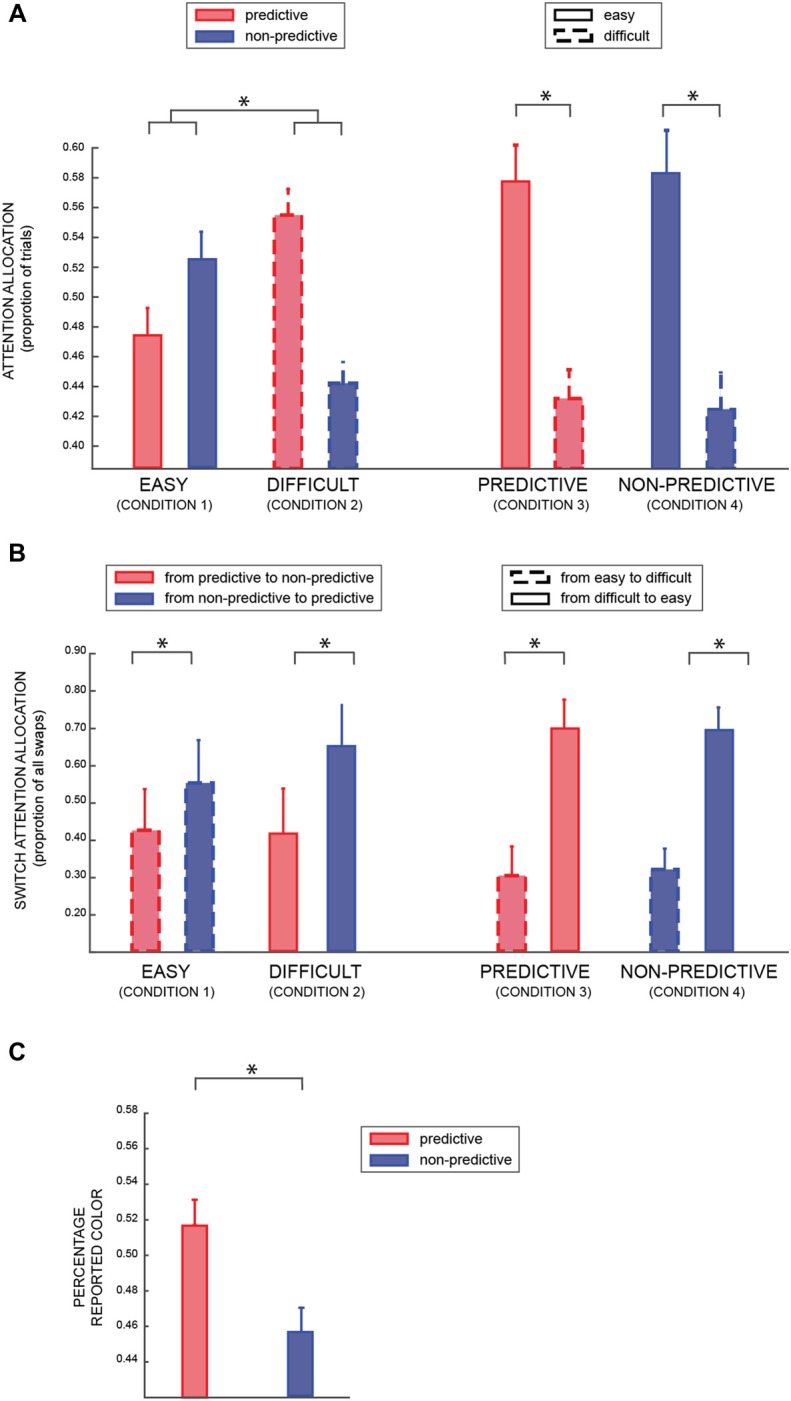
**(A)** The average percentage of trials in which participants looked at predictive or non-predictive patches (conditions 1 and 2—see Table 1), respectively red and blue (left part); and high or low coherence level (conditions 3 and 4), respectively solid and dashed lines (right part). **(B)** Average percentage of trials in which participants switched attention from one patch to the other (left column: from predictive to non-predictive and vice versa –conditions 1 and 2; right column: from low to high coherence level and vice versa—conditions 3 and 4). **(C)** Average percentage of time participants reported the predictive (red) or non-predictive (blue) color. In all the panels, error bars are standard errors, and asterisks indicate significant difference (*p* < 0.05).

For the eye movement analysis, we considered as binary dependent variable the proportion of trials in which participants attended the predictive or the non-predictive patch (Table 1: conditions 1 and 2; model Va), when both patches were either easy or difficult (factors PREDICTABILITY and DIFFICULTY respectively). As shown in Figure 3A, we found a significant result for both factors (DIFFICULTY BF_10_ = 11.48, PREDICTABILITY BF_10_ = 18.37, positive evidence), and a strong interaction between the two factors (BF_10_ = 53.61, strong evidence). A *post hoc* analysis revealed a significant difference between predictable and non-predictable colors in the difficult (BF > 100) but not in the easy condition (BF = 1.433), suggesting that participants were looking more at the informative patch when the coherence of both patches was lower (i.e., 25%), in agreement with the results of the previous analysis (model III).Not surprisingly, in the condition in which one of the patches was easy and the other one difficult (Table 1: conditions 3 and 4; model Vb), we found a very significant effect of the factor DIFFICULTY (BF_10_ ≫ 100).

Figure 3B shows the proportion of trials in which participants made a saccade from one patch to the other (around 15% of trials, with three participants having a percentage closer to 40% and all the other participants <10%). We found a positive effect of PREDICTABILITY (left part of the Figure; BF_10_ = 5.11, positive evidence), irrespective of the difficulty level of both patches (Table 1: conditions 1 and 2; model VIa). Similarly, in conditions 3 and 4 (model VIb), we found a significant effect of the difficulty level (right part of the Figure; BF_10_ > 100).

Finally, we investigated which color was reported more frequently when participants were asked to report the patch’s color in 10% of the trials. Importantly, in these trials one patch had a predictive color and the other patch a non-predictive one, so it provided us with an indirect measure of attentional allocation. Considering only trials in which participants reported a color that had actually been displayed (accuracy for this task was >95%), we found that participants reported more frequently the predictive than the non-predictive color (Bayesian paired *t*-test of choosing the predictive color against chance level: BF > 40, very strong evidence), as shown in Figure 3C, in agreement with the previous analyses that suggested a bias of attention toward the informative colors.

## Discussion

In this study, we investigated the effect of unconscious learning on visual attention by means of eye movements, and how unconscious biases are influenced by conscious, task-relevant information (i.e., signal strength). Participants were instructed to report the motion direction of two patches of variable coherence and color: unbeknown to them, two out of three colors were 100% informative of the correct response. Participants failed to notice this association consciously, but nevertheless exploited the color information to perform the task, replicating our previous findings (Alamia et al., 2016). The question we addressed in the current study was whether unconscious knowledge of the color-motion association affects eye movements, and how this influence interacts with the strength of the stimuli (i.e., coherence of the patch). As expected, we found that the color cue affected behavioral measures (RT and accuracy), and that participants were attending more frequently the patches exhibiting the predictive colors, despite not being consciously aware of the associations. Importantly, this pattern occurred more frequently during difficult trials, that is when the motion direction was harder to perceive, thus revealing an interaction between conscious and unconscious features of the stimuli in biasing attentional allocation.

One crucial point of this study is the measure of awareness of the association: we rely on a four-item questionnaire, in which the last question directly probes the knowledge of the color-motion association. In our previous study (Alamia et al., 2016), we investigated awareness of the same association in a simpler design, with only one patch per trial and fixed coherence. We showed that participants who failed the questionnaire also failed more sensitive tests (i.e., generative and familiarity tests- Derosiere et al., 2017). This indicates that -in this task- the questionnaire provides a reliable measure of awareness, and that other tests would have reached similar conclusions. Moreover, it is legitimate to assume that participants whom are aware of the color-motion associations would base their choices to a large extent on color, leading to different pattern of results, with drastic differences between conditions (as reported in Alamia et al., 2016). Yet, we did not observe such effects in our results. Altogether, these considerations provide good evidence in favor of truly unconscious bias of color on decisions.

Several previous studies have investigated the relationship between attention and unconscious learning. On the one hand, recent studies have investigated how attentional allocation is affected by unconscious sources of information. One study from Zhao et al. (2013) investigated the relationship between statistical learning and visual attention (i.e., spatial and feature-selection). In a series of experiments, one out of four locations displayed a statistically structured sequence of abstract shapes, whereas the order was random in the other locations. They showed that RTs of an orthogonal task were decreased in the location exhibiting statistical regularities, thus suggesting that covert attention was affected by regularities even when these were not relevant for performing the task. Interestingly, another study about visual selection suggested that attention was driven away from the location where distractors were more likely to appear (Wang and Theeuwes, 2018). It is note worthy, however, that in that study some participants—yet included in the analysis—reported to have explicitly noticed the regularity.

On the other hand, several studies have also investigated the reciprocal relationship: how attentional allocation affects the implicit learning of statistical regularities. Regarding visual statistical learning, a seminal study from Turk-Browne and colleagues (Turk-browne et al., 2005) has revealed that selective attention is necessary to implicitly learn the regularities of a stream of stimuli: in his study the unattended stimuli were not learnt by the participants. Conversely, a recent study failed to replicate these findings, showing that the unattended regularities are learnt as well as the attended ones (Musz et al., 2015). Besides visual statistical learning, the impact of attention on unconscious learning has been also investigated in the context of implicit sequence learning. In a serial RT task (SRTT), the most commonly used paradigm to study implicit sequence learning, participants learn a sequence of responses implicitly, and these sequences can be either deterministic, probabilistic or random. In the first two cases (i.e., when the sequence is predictable) participants perform better than in the random case, even when they are not aware of the predictability (Destrebecqz and Cleeremans, 2001; Destrebecqz et al., 2005). In a seminal study, Nissen and Bullemer (1987) showed that the addition of a secondary orthogonal task, which pulls participant’s attentional resources away from the main task, impaired learning, thus indicating the crucial role of attention in implicit learning; whereas some other authors suggested a rather different interpretation, framing the results in terms of interference between the first and the secondary task (Stadler, 1995), and not in terms of attentional resources. Conversely, Cohen et al. (1990) showed no effect of a secondary task on participants’ performance, even though further studies failed to replicate these results (Frensch et al., 1998). Finally, the results of Cleeremans et al. (1998) fell in between, showing lesser but significant implicit learning in the presence of an orthogonal task. All in all, the role of attention in implicit learning in the context of SRTT remains disputed.

Beyond implicit learning, a few studies have investigated how subliminal stimuli affect attentional allocation, and conversely how subliminal stimuli are affected by visual attention. Specifically, one study from Kanai et al. (2006) investigated how attentional allocation affects subliminal perception. In their task, the perception of the orientation of a grating pattern was altered by the presentation of a previous stimulus, which was allegedly subliminal due to continuous flash suppression. This tilt after effect (TAE) was induced by a subliminal stimulus (due to continuous flash suppression) at two different locations, one of which was attended by the participants. Their results showed a TAE with subliminal adaptors both when participants were attending the location and when they were not, suggesting that spatial attention does not influence low level processing of subliminal stimuli (Kanai et al., 2006). Regarding the effect of subliminal stimuli on attentional allocation, other studies have showed that spatial attention can be affected by subliminal stimuli (Mulckhuyse et al., 2010; Chou and Yeh, 2011; Mastropasqua and Turatto, 2015).

All in all, the results from the current literature seem to suggest that unconscious information, both subliminal and supraliminal, can affect visual attention. However, the actual unconscious nature of these processes has been strongly questioned (Shanks, 2003; Smyth and Shanks, 2008; Vadillo et al., 2016). In this regard, this study adds an important and original contribution to the previous literature. As such, we used a paradigm whose rules are simple to learn and in which all relevant information is supraliminal (i.e., color and motion coherence), thus voiding possible confounds related to other implicit learning paradigms (Shanks and St. John, 1994; Newell and Shanks, 2014). As discussed at length in our previous study (Alamia et al., 2016), the simplicity of the unconscious association ensures that participants do not exploit alternative strategies to perform the task (as in more complex paradigms, e.g., artificial grammars). Additionally, the usage of supraliminal stimuli allows us to avoid the issues related to subliminal perception, since in that domain it is difficult to determine whether stimuli are truly unconsciously perceived or not (Lovibond and Shanks, 2002).

The conclusion that attention is biased toward the most informative patch is in line with our previous findings that suggested that unconscious information bias attentional allocation only when it is beneficial for task performance (Alamia and Zénon, 2016). Whereas in that earlier study, we manipulated the relevance of the unconscious information, here we manipulated the strength of the conscious source of information. These combined findings suggest that attentional allocation to unconscious cues depends on top-down mechanisms since it is modulated by high-level factors, such as relevance and relative reliability.

Further than that, another important element of novelty introduced in our study lays in the interaction between unconscious and conscious sources of influence on attentional allocation. An intriguing perspective raised by this finding is that unconscious and conscious attentional cues are weighted as a function of their behavioral reliability, as suggested by the fact that attention is deployed to the most informative stimuli primarily when both patches have low coherence (i.e., when the task is more difficult). Importantly, the unconscious color-motion association was as effective in the easy as in the difficult trials, indicating that failure to allocate attention to predictive stimuli in the high-coherence trials was not caused merely by the fact that subjects did not use the unconscious information in easy trials. Additionally, this interpretation would utterly fit within a Bayesian framework, in which cues are weighted based on the variance of their distribution (i.e., precision, Feldman and Friston, 2010), as much as sensory cues from differences modalities are combined based on their reliability (Battaglia et al., 2003). However, further experiments are required to effectively test this hypothesis.

As suggested in other studies (Beilock et al., 2002; Olivers and Nieuwenhuis, 2005), attention and consciousness can have different and dissociable effects on behavior, thus hinting that these two processes potentially rely on different neuronal mechanisms. Our results advocate in favor of the dissociation between attention and consciousness (Koch and Tsuchiya, 2007, 2012; Tsuchiya and Koch, 2016), showing that it is possible to have an attentional effect (i.e., eye movement) which is driven by unconscious information: conceivably, participants are aware of which patch they are attending at each trial, but their choice is influenced to some extent by unconscious knowledge. In conclusion, our study shows that unconscious processing affects attentional allocation and eye movements in a perceptual decision-making task.

## Author Contributions

AA and AZ conceived the study and contributed to the interpretation of data. AA realized the experiments. AA and OS processed the data. All the authors wrote the manuscript.

## Conflict of Interest Statement

The authors declare that the research was conducted in the absence of any commercial or financial relationships that could be construed as a potential conflict of interest.
